# Individual Importance Weighting of Domain Satisfaction Ratings does Not Increase Validity

**DOI:** 10.1525/collabra.116

**Published:** 2018-02-26

**Authors:** Julia M. Rohrer, Stefan C. Schmukle

**Affiliations:** * Department of Psychology, University of Leipzig, Leipzig, DE; † International Max Planck Research School on the Life Course (LIFE), Max Planck Institute for Human Development, Berlin, DE; ‡ German Institute for Economic Research (DIW Berlin), Berlin, DE

**Keywords:** well-being, life satisfaction, domain satisfaction, domain importance, weighting

## Abstract

Bottom-up models of life satisfaction are based on the assumption that individuals judge the *overall* quality of their lives by aggregating information across various life domains, such as health, family, and income. This aggregation supposedly involves a weighting procedure because individuals care about different parts of their lives to varying degrees. Thus, composite measures of well-being should be more accurate if domain satisfaction scores are weighted by the importance that respondents assign to the respective domains. Previous studies have arrived at mixed conclusions about whether such a procedure actually works. In the present study, importance weighting was investigated in the Panel Study of Income Dynamics (PSID; *N* = 5,049). Both weighted composite scores and moderated regression analyses converged in producing the conclusion that *individual* importance weights did not result in higher correlations with the outcome variable, a global measure of life satisfaction. By contrast, using weights that vary *normatively* across domains (e.g., assigning a larger weight to family satisfaction than to housing satisfaction for all respondents) significantly increased the correlation with global life satisfaction (although incremental validity was rather humble). These results converge with findings from other fields such as self-concept research, where evidence for individual importance weighting seems elusive as best.

What happens in people's heads when they are asked how satisfied they are with their lives? So-called bottom-up models are based on the assumption that people assess the conditions of their lives across various life domains and then aggregate these pieces of information to arrive at their judgments (e.g., Lucas, 2004). Beginning with the seminal work on life satisfaction by Campbell, Converse, and Rodgers (1976), the idea that this aggregation takes into account weights that vary between respondents has been popular. These weights might reflect respondents' personal values (Oishi, Diener, Suh, & Lucas, 1999) and can potentially be influenced by factors such as the surrounding culture (Oishi, Diener, Lucas, & Suh, 1999) or respondents' age (George, Okun, & Landerman, 1985).

Such a weighted bottom-up model has a strong intuitive appeal: Surely, it makes sense to expect that somebody who cares a lot about his or her family will be less satisfied with his or her life in general when family life is unsatisfactory, whereas somebody who does not care about relatives would probably not be affected too much. A similar importance-weighted model has been popular in self-esteem research and can be traced back as far as William James' “The principles of psychology” (see Marsh, 2008), illustrating the high face validity of such models. In addition, the application of a weighted bottom-up model opens the door to an appealing individualized approach to the measurement of life satisfaction: What is most important to respondents should carry the largest weight in a composite score for assessing their quality of life.

*In theory*, such an idiographic approach is fairly straightforward. Respondents report (a) how satisfied they are with the central domains of their lives and (b) how important these domains are, either by ranking them or by assigning ratings. Researchers can then weight the satisfaction ratings by the importance ratings to arrive at a personalized composite score of overall life satisfaction. Such a measure should be a more valid reflection of how respondents' lives are going than a simple sum score, which represents the implicit assumption that each domain is equally important—in order words, a sum score is equal to applying uniform weights to all domains. The weighted composite score can then be used to predict other variables. Most studies on this topic have relied on correlations between the weighted composite score and a global measure of life satisfaction to gauge the validity of the weighted score. If the weighted composite score shows a higher correlation than an unweighted sum score, this is interpreted as evidence that the importance weighting hypothesis has merit.

However, *empirically* speaking, studies have frequently failed to find evidence that importance weighting results in improved validity—including Campbell et al., themselves (1976, pp. 86–93), followed by others. For example, Philip, Merluzzi, Peterman, and Cronk (2009) found that a weighting algorithm did not improve a measure of health-related quality of life in a sample of 194 cancer patients. Likewise, Russell, Hubley, Palepu, and Zumbo (2006) found that weighted scores from the Injection Drug User Quality of Life Scale did not outperform unweighted scores in a sample of 241 adults. Wu (2008) also found that weighted scores were not superior to unweighted scores in a sample of 167 undergraduates. Note that all these sample sizes were rather small, and the statistical power these studies had to find evidence for the superiority of the weighting procedure was quite low when considering that importance weighting effects should show up as between-subject interactions.

Trauer and Mackinnon (2001) argued that such multiplicative weighted scores are “undesirable and unnecessary” (p. 579). Their first point (i.e., the scores are undesirable) addresses the idea that the reliability of weighted sum scores is somewhat questionable. To offer an intuitive explanation for this: Both satisfaction and importance are not measured perfectly, and thus, the multiplication of these two already flawed measures might exacerbate measurement error. On top of this, Trauer and Mackinnon claimed that weighted average scores are hard to interpret and sensitive to the scaling of composites. For example, results are not invariant under a simple linear transformation of importance ratings.^1^ Their second point (i.e., the scores are unnecessary) addresses the idea of weighted models in general: Domains incorporated into questionnaires have already been selected to be universally relevant, and thus, they should be important to almost all respondents. Importance weighting should therefore be unnecessary.

Moderated regression analyses provide another way to assess importance weighting that is built on less strict assumptions and provides a test that is insensitive to the scaling of the importance ratings (i.e., arbitrary linear transformations of the scale do not affect the conclusions). In moderated regression analyses, the outcome (usually a global measure of life satisfaction) is predicted from satisfaction ratings, importance ratings, and their interactions. A positive interaction between a person's satisfaction in a specific domain and the respective importance rating is interpreted as evidence for the merit of importance weighting. In comparison with composite scores, moderated regression analyses distinguish between the main effects of satisfaction and importance ratings and their interactions, whereas weighted composite scores result in one estimate in which these sources of variation are intertwined.

Wu and Yao (2006) claimed to have found evidence for the merit of importance weighting in such moderated regression models using a sample of 130 undergraduate students, but once again, this sample size seems fairly small for reliably assessing the evidence for between-subject interactions. Tiefenbach and Kohlbacher (2015) analyzed a larger sample of 2,900 Japanese adults and arrived at a more qualified conclusion that importance ratings moderated the association between domain satisfaction and happiness in some but not all domains. Note that it is close to impossible to actually interpret the estimates from their moderated regression analysis because the model included satisfaction with “purpose in life (regarding work, hobbies, and social contributions)” as a predictor of the outcome, which was happiness. This is arguably less of a life domain and more an alternative potential outcome measure, and judging from the results reported in the paper, it obscured any association between the other life domains (e.g., finances, health, family) and happiness.

Hsieh (2003, 2012) put forth multiple arguments for weighting by domain importance, claiming that weighting works when domains are ranked from most to least important instead of being rated on separate rating scales. Both studies were based on the same data from 90 telephone interviews of respondents aged 50 years or older, but again, this sample might be considered underpowered. An additional study by Hsieh (2016) used latent class analysis and cluster analysis to identify different patterns of importance, then argued that the relationship between global life satisfaction and a domain-satisfaction composite varied between groups. Whereas this study used a larger sample (*N* = 2,164; note that this is the same sample as used in Campbell et al., 1976, who did not find evidence for importance ratings as weighting factors), the analytical approaches at best provide a *very* indirect test of importance weighting.

Lastly, Marsh and Scalas (2017) developed a taxonomic SEM approach, which is conceptually comparable to the moderated regression approach but also incorporates latent factors, to test individually weighted-average models and included a quality of life measure as one of their empirical illustrations. Even though the focus of their article is on methodological matters rather than well-being, it might well constitute the most rigorous test of importance weighting and life satisfaction to date, including a sample of 2,751 respondents in their mid-twenties and employing a systematic SEM approach. Overall, the authors concluded that the interactions between satisfaction and importance explained only very little unique variance, providing only very limited support that individuals do in fact apply importance weighting in their quality of life judgments.

Taken together, the current state of the literature does not allow for a clear and straightforward judgment to be made about importance weighting. Results are difficult to integrate across studies because different authors used different methodological approaches (weighted scores, moderated regression analyses, the complex classification procedure used by Hsieh, 2016, and the SEM approach by Marsh & Scalas), included variable and sometimes questionable selections of “life domains” that rendered the results uninterpretable (e.g., “purpose in life satisfaction”), and investigated effects in very different samples (e.g., undergraduates from Taiwan vs. individuals aged 50 or older in Chicago), most of them not even remotely representative of any general population that a researcher might be interested in. In addition, the sample sizes have frequently been surprisingly small given that the effects of interest are between-subject interactions, and thus, the extent to which we know anything about the validity of importance weighting remains unclear.

## The Present Study

In this study, we investigated importance weighting in a large-scale sample of the U.S. adult population: Does the effect of satisfaction in a certain life domain on global life satisfaction vary by the importance assigned to the respective domain?

As preparatory work, we first analyzed the basic characteristics of domain importance and domain satisfaction items because these would determine whether an empirical importance weighting approach is promising to begin with. For example, if all respondents agreed that a certain domain was important (or unimportant), then there would actually be no need to collect importance ratings. In addition, we tested the relationship between domain importance and domain satisfaction. Many researchers conducting studies investigating importance weighting have implicitly assumed that these two types of ratings are orthogonal, which would admittedly render the weighting procedure somewhat more conceptually “elegant.” Thus, the researchers who conducted such studies did not report whether they found a correlation between the importance and satisfaction ratings. However, if the importance of a specific domain was strongly correlated with satisfaction with that domain, the information provided by the two items would become redundant.

To test the central hypothesis of importance weighting, we applied weighting procedures and moderated regression analyses, and we discuss the methodological properties of both approaches. We elaborate on the crucial distinction between normatively as compared to individually weighted scores, an issue that has caused lots of confusion and false claims in the literature. At last, we report additional exploratory analyses to test whether importance weighting is relevant in only some domains.

## Method

### Initial Sample

Data came from the well-being supplement of the Panel Study of Income Dynamics (PSID) 2016. The PSID is a nationally representative panel survey of families in the United States, produced and distributed by the Survey Research Center at the University of Michigan. In 2016, a questionnaire on well-being (PSID-WB; Freedman, 2017) was added to the study. This was a brief self-administered instrument completed via the internet or on paper. The supplement covered various measures of well-being, personality, and activities, as well as ability measures.

Heads of household and their partners who were at least 30 years old by December 31, 2015 were eligible for the PSID-WB in 2016. Eligible panel members were mailed an invitation letter including the web address of the survey and login credentials and received a $20 check upon completing the survey. Of the 10,689 eligible cases, 8,341 responded (78% response rate). Age ranged from 30 to 97, *M* = 50.55, *SD* = 14.37. Women comprised 56.35% of the sample.

### Assessment of Global Life Satisfaction

Respondents answered the Satisfaction With Life Scale (SWLS; Diener, Emmons, Larsen, & Griffin, 1985), one of the standard measures in well-being research consisting of five items (e.g., “In most ways, my life is close to my ideal”) rated on a 5-point response scale (1 = *strongly disagree*, 2 = *somewhat disagree*, 3 = *neither agree nor disagree*,4 = *somewhat agree*, 5 = *strongly agree*). The scale had a satisfactory reliability of α = .89. Due to missing responses to SWLS items, 160 respondents had to be excluded.

### Assessment of Domain Importance

Respondents reported the importance of 10 different life domains on a 5-point response scale (0 = *not at all important*, 1 = *a little important*, 2 = *somewhat important*, 3 = *very important*, 4 = *extremely important*).^2^ The phrasings of the items as well as domain abbreviations that will be used throughout the manuscript can be found in Table 1.

### Assessment of Domain Satisfaction

Respondents reported their satisfaction with 10 different life domains on a 5-point response scale (0 = *not at all satisfied*, 1 = *a little satisfied*, 2 = *somewhat satisfied*, 3 = *very satisfied*, 4 = *completely satisfied*). These 10 items corresponded to the 10 importance items (see Table 1). Note that for the satisfaction items, an additional response option, *Does not apply to me*, was available because not all items (e.g., job, marriage/relationship, faith) applied to all respondents. We restricted analyses to all respondents who answered all 10 domain satisfaction items. Thus, the final sample consisted of *N* = 5,049 respondents (54.03% women) with a mean age of 46.82 years (*SD* = 12.13). Notice that because of this considerable sample size, even small effects can reach the conventional threshold for statistical significance. In the results section, we will consider changes in *R*^2^ to evaluate whether importance weighting is able to account for meaningful amounts of variance in the outcome variable.

### Software

All analyses were run in R (R Core Team, 2017) using the RStudio environment (RStudio Team, 2016) and a number of R packages. More precisely, dplyr (Wickham & Francois, 2016), tidyr (Wickham, 2016), and reshape2 (Wickham, 2007) were used for data wrangling; psych (Revelle, 2016) was used for the Fisher *z*-transformation and to compare the correlation coefficients; lavaan (Rosseel, 2012) was used to fit the regression model with constrained coefficients; and mlr (Bischl et al., 2016) was used for cross-validation. All plots were generated with the help of ggplot2 (Wickham, 2009).

## Results and Discussion

### Mean Scores and Variances of Domain Importance and Satisfaction

For all of the 10 domains, the mean importance ratings were high, exceeding a mean response of 3 (*very important*; see Table 2). By contrast, the mean satisfaction ratings were not that close to the upper end of the response scale but were still fairly high: All mean scores fell above the middle response option of 2 (*somewhat satisfied*; see Table 3).

Given the high mean scores, it was no surprise that variability in the importance ratings was generally low. For example, 71.52% of respondents reported that their family was *extremely important* to them, and 25.85% rated their family life as *very important*, whereas only 0.42% used one of the two lowest response options (*a little important*, *not at all important*). The domain that showed the highest variability in importance, faith, still showed considerable concentration at the high end of the scale: 49.97% reported that having a strong religious faith was *extremely important* to them, and 25.27% rated it *very important*, whereas 9.88% of respondents chose one of the options at the lower end of the scale (a little important, not at all important).

Domain satisfaction scores consistently showed somewhat higher variability for all domains except for faith, although the responses were still concentrated in the upper half of the scale. For example, 35.75% of respondents reported that they were *completely satisfied* with their family life, and 41.59% were *very satisfied*, whereas only 5.17% used one of the two response options at the lower end of the scale (*a little satisfied*, *not at all satisfied*).

Taken together, both the domain importance and domain satisfaction ratings tended to be located in the upper half of the response scales. In particular, domain importance items tended to be answered with the two highest response options and accordingly, variability in those items was limited.

### The Relationship between Domain Importance and Domain Satisfaction

Are respondents more or less satisfied with domains that are more important to them? This question could be approached from two perspectives.

From a variable-centered perspective, it would make sense to ask: Are respondents who rate health as more important *compared with other respondents* more/less satisfied with their health *compared with other respondents*? This question can be answered by investigating bivariate correlations between domain importance and domain satisfaction, and this analysis would result in one estimate per domain (across all respondents).

From a person-centered perspective, it would make sense to ask: Are respondents who rate health as more important *compared with other domains* more/less satisfied with their health *compared with other domains*? This question could be answered by investigating profile correlations between domain importance ratings and domain satisfaction ratings, and this analysis would result in one estimate per respondent (across all domains). Note that the two approaches are intrinsically related on a statistical level (see Allik et al., 2015).

#### Variable-centered perspective

**Figure 1:**
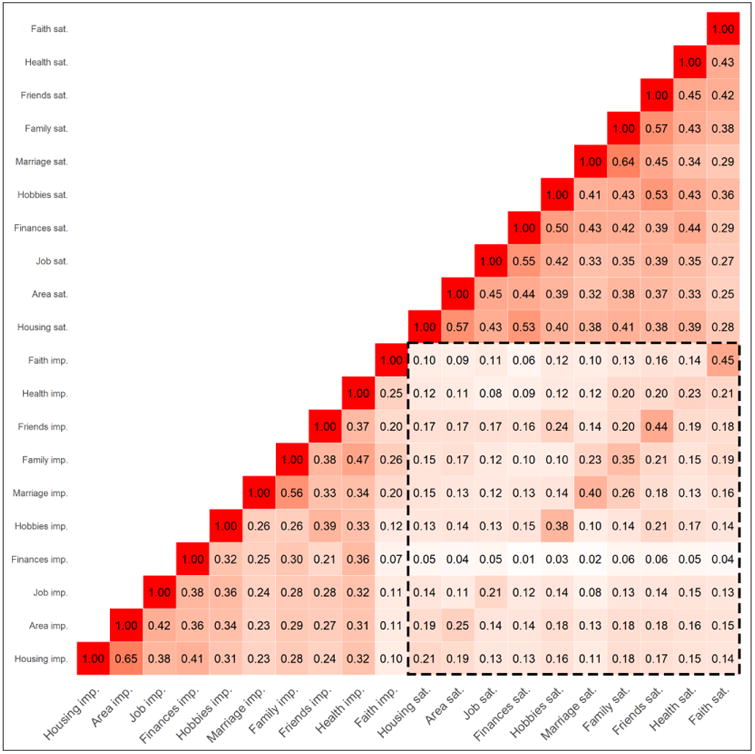
Intercorrelations between all domain importance and domain satisfaction items. *N* = 5,049; *r*s exceeding .03 are significant at *p* < .05, *r*s ≥ .04 at *p* < .01, and *r*s ≥ .05 at *p* < .001.

For all domains except for finances, there was a positive correlation between domain importance and domain satisfaction. Respondents who rated domains as more important also said they were more satisfied with these domains. This can be seen in Figure 1, which depicts the correlations between all importance items and all satisfaction items. Of interest at this point is the diagonal within the highlighted square in the bottom right corner of the figure. These correlations ranged from *r* = .21 (housing, job) to .45 (faith), all *p*s < .001, with the exception of the domain finances that showed quite a distinct pattern with virtually no correlation between the importance of finances and satisfaction with finances (*r* = .01, *p* = .473): Respondents who gave high ratings to the importance of financial security were not more or less likely to report being satisfied with their financial situation.

This positive association for the nine other domains *could* reflect a general tendency to report high importance and high satisfaction—respondents might have a certain tendency to reply with high or low values on the rating scales, and this might result in the appearance that satisfaction and importance are correlated for each of the domains. However, these correlations were specific to the domains to some extent, ruling out the possibility that this alternative explanation could completely account for the pattern. Although there were some substantial correlations across domains (e.g., the importance of friends was correlated *r* = .24 with satisfaction with hobbies; see Figure 1), the correlations linking importance and satisfaction for the same domain always numerically exceeded the cross-domain correlations except for the domain of finances (which was an exception to begin with, since it did not show a correlation between importance and satisfaction ratings).

Note that using Spearman's rank order correlation instead of Pearson's correlation coefficient changed the specific numbers only slightly but did not affect the pattern at all; see the additional figure provided on the OSF project page, https://osf.io/m3ezs/.

#### Person-centered perspective

For each respondent, a profile correlation coefficient was calculated by correlating the 10 importance items with the 10 corresponding satisfaction items. Correlations were Fisher *z*-transformed *before* averaging, and *afterwards*, they were transformed back into the more common correlation metric. The average profile correlation coefficient between domain importance and domain satisfaction was *r* = .34 and was significantly different from zero, *t*(4,210) = 29.89, *p* < .001. As a robustness check, we standardized each variable separately and then recalculated the profile correlations. This procedure ensured that the individual profile correlations were not confounded by normative patterns (i.e., correlations between the mean importance ratings and the mean satisfaction ratings *across respondents*). Again, the average profile correlation coefficient (*r* = .30) was positive and significantly different from zero, *t*(5,048) = 45.77, *p* < .001.^3^

Taken together, importance and satisfaction were mildly correlated both across and within respondents. Respondents who considered a domain more important were also more satisfied with that domain. Furthermore, on average, if a respondent considered a specific domain more important than other domains, he or she was also more satisfied with that domain than with other domains. However, all these correlations were moderate in magnitude, so that there is no reason to assume that importance and satisfaction ratings are essentially redundant.

### Incremental Validity in Predicting Global Life Satisfaction: Weighting

Does weighting by importance increase the amount of variance that domain satisfaction ratings can explain in a measure of global life satisfaction? Previous studies have frequently calculated importance-weighted composite scores and have subsequently compared the correlation between these weighted scores and simple (unweighted) sum scores.

To arrive at an individually weighted composite score, each domain satisfaction rating for each respondent is multiplied by the respective domain importance rating, all these products are summed, and the sum is divided by the number of all importance ratings made by the respective respondent, that is: $\text{Individually weighted composite}=\frac{\sum ({\text{Satisfaction}}_{i}*{\text{Importance}}_{i})}{\sum ({\text{Importance}}_{i})}$ with *i* indexing the different life domains.

This individually weighted composite was highly correlated with the simple sum score of all domain satisfaction ratings (i.e., the unweighted composite; *r* = .993, *p* < .001, see Table 4). Thus, it was no surprise that the correlations between the two different composites and the SWLS score were of almost exactly the same magnitude: Whereas the correlation between the unweighted composite and the SWLS score equaled *r* = .695 (*R*^2^ = 48.37%), the correlation between the individually weighted composite and the SWLS score was only slightly higher, *r* = .699 (*R*^2^ = 48.86%).

### Disentangling Normative and Individual Weighting

Note that there are two potential (noncompeting) explanations for the incremental validity of the individually weighted composite.

First, the added validity might stem from a *normative* importance effect. For example, respondents might collectively assign higher weight to a domain that is indeed more strongly related to global life satisfaction across respondents (e.g., family satisfaction in our data). In a regression model predicting global life satisfaction from only the domain satisfaction scores, this would show up as different regression coefficients for the various domain satisfaction ratings. Such a normative importance effect would make it superfluous to collect individual importance ratings if the researcher knew which domains the average respondent considered more important.

Second, the added validity might stem from *idiosyncratic/individual* importance effects. For example, a respondent who assigns a higher weight to a specific domain might be more strongly influenced by this specific domain, *regardless* of the importance assigned to this domain by other respondents. In a regression model predicting global life satisfaction from domain satisfaction, domain importance, and their interaction, this would be indicated by a significant interaction effect. In order to account for such individual importance effects, one would have to collect individual importance ratings.

This distinction between normative and individual weighting has played a crucial role in discussions about importance weighting in self-esteem research (Marsh, 2008; Marsh & Scalas, 2017) and is equally relevant when it comes to importance weighting in well-being research, as the substantive interpretation differ substantially: Only individual weighting supports the common notion that the *individual* applies importance weights when forming summative judgments.

To assess the evidence for the two different types of weighting within the multiplicative weighting framework, we additionally generated a weighted composite score in which the individual domain satisfaction ratings were multiplied by the average importance rating *across respondents*. This normatively weighted composite, $\text{Normatively weighted composite}=\frac{\sum ({\text{Satisfaction}}_{i}*\text{Mean}({\text{Importance}}_{i}))}{\sum (\text{Mean}({\text{Importance}}_{i}))}$, assigns different weights to different domains; however, the domain-specific weights are the same for all respondents.

This normatively weighted composite was highly correlated with both the unweighted composite (*r* = .9997, *p* < .001) and the individually weighted composite (*r* = .993, *p* < .001). Its correlation with the SWLS score was comparable to the other two composites (*r* = .698, *R*^2^ = 48.74%), and it significantly outperformed the unweighted composite according to Steiger's test (*t* = 11.49, *p* < .001; Steiger, 1980) in predicting the SWLS score (an additional 0.38% of explained variance). However, the individually weighted composite score did *not* significantly outperform the normatively weighted composite score, *t* = 0.68, *p* = .490. According to these numbers, it seemed sensible to prefer the more parsimonious model in which only one weight was assigned to each domain across all respondents instead of a model that was based on the assumption that there are interindividual differences in the weights.

### Incremental Validity in Predicting Global Life Satisfaction: Moderated Regression

As outlined above, an alternative approach to assessing whether the effects of domain satisfaction on global life satisfaction are weighted by domain importance uses moderated regression analyses. To highlight both the commonalities and the discrepancies between the two statistical approaches, we ran a sequence of regression models to lead up to the key model that we used to test for the incremental validity of the importance weighting procedure.

In the initial model (Model 0), the SWLS score was predicted from the 10 satisfaction ratings, and all coefficients were constrained to equality. This model is based on the assumption that each of the 10 domains is equally important for life satisfaction. Estimating this model yielded exactly the same results as correlating the unweighted composite (i.e., the simple sum score of all 10 items) with the SWLS score. Thus, it was no surprise that in this model, *R*^2^ = 48.37% of the variance in the SWLS score could be explained; this equals the square of the correlation between the unweighted composite and the SWLS (see above).

In the next step, the SWLS score was predicted from the 10 domain satisfaction ratings, but the coefficients of the predictors were allowed to vary as in regular linear regression analyses. This conceptually corresponds to the normatively weighted score above. All in all, *R*^2^ = 51.63% of the variance in the SWLS score was explained by the predictors (coefficients in Table 5, column Model 1). However, it would be unfair to compare this number with the performance of the composite scores or the regression model with fixed coefficients: Because the coefficients for each life domain are estimated from the data, the model becomes flexible. Thus, this model will necessarily provide a better fit to the data at hand than the composite scores or the regression with fixed coefficients, but this does not necessarily generalize to new samples from the same population because the regression might overfit sample-specific noise (for an accessible introduction to the problem of overfitting, see Yarkoni & Westfall, 2017). Therefore, we additionally evaluated the performance of this regression model using 10-fold cross-validation. This resulted in a slightly lower performance of *R*^2^*_cross-validated_* = 51.14%. Thus, it might be more realistic to estimate that 51.14% – 48.37% = 2.77% of the variance could be explained when allowing the weights of the domains to vary. Note that this model conceptually mirrors the normatively weighted composite score described above: The weights are allowed to vary between domains, but for each domain, they are the same across all respondents. However, in the case of the normatively weighted composite score, the weights for the domains are derived from the average importance ratings. In the regression model described here, they are estimated from the data and informed by the outcome, the SWLS score. The regression model provided a better fit to the data than the normatively weighted composite score (R^2^ = 48.49% vs. R^2^_cross-validated_ = 51.15%), indicating that it might be preferable to estimate the weights assigned to the domains from the data instead of based on the importance ratings, although the difference was rather small.

The next model additionally incorporated the *main effects* of the 10 domain importance ratings. In this model, again, slightly more variance in the outcome could be explained (*R*^2^ = 51.94%, *R*^2^*_cross-validated_* = 51.22%, coefficients in Table 5, column Model 2), and a model comparison test indicated that this difference was statistically significant, χ^2^(10) = 11.178, *p* < .001. Thus, the domain satisfaction importance ratings themselves were seemingly able to explain some variance in the global life satisfaction measure, but the gain was negligible.

The key regression model that was computed to test the importance weighting hypothesis additionally incorporated the interaction between the domain satisfaction ratings and the corresponding domain importance ratings. Thus, the outcome (SWLS score) was predicted from the 10 domain satisfaction ratings, 10 domain importance ratings, and 10 interactions. In this model, again, slightly more variance was explained (*R*^2^ = 52.20% or 0.26% more than the model without interaction terms, coefficients in Table 5, column Model 3) in the sample, and a model comparison indicated that this was statistically significant, χ^2^(10) = 9.08, *p* = .003. However, results from the cross-validation procedure indicated that this model was not really preferable to the previous model, *R*^2^*_cross-validated_* = 51.23%. When comparing the models without cross-validation, the interaction terms seemed to contribute to the prediction of the outcome—but the performance of the two models was virtually identical when applied to new data points from the same population (Δ*R*^2^ = .00009, or 0.009%), suggesting that the in-sample performance of the more complex model might be an overestimation due to an overfitting of the data at hand. Thus, results from the moderated regression analyses did not support the idea of individual importance weighting.^4^

### Alternative and Additional Analyses

To further explore the data—and to rule out that we failed to find support for importance weighting because we chose the wrong analytic approach—we ran several additional analyses.

#### Moderated regression with within-subject centered weights

We modified the moderated regression approach to be closer aligned with a person-centered perspective. For that purpose, we centered the importance ratings *within* subjects so that they express relative importance of a domain compared to the other domains. We then used these within-subject centered importance ratings in a moderated regression as described above. The performance of this model (*R*^2^ = 52.24%, *R*^2^*_cross-validated_* = 51.22%) was virtually identical to the performance of the moderated regression model without within-subject centering described above (*R*^2^ = 52.20%, *R*^2^*_cross-validated_* = 51.23%) and thus also failed to support the idea that the importance * satisfaction ratings play a large role.

#### Including more respondents but less domains

Seven of the domains (Housing, Area, Finances, Hobbies, Family, Friends, Health) are arguably relevant to almost all respondents, regardless of their living circumstances. We thus repeated the central moderated regression analyses only including those seven domains, which allows for a considerably larger sample size (*N* = 7,439, 56.27% female, *M_age_* = 49.79, *SD_age_* = 13.92). In Model 0 (predicting the SWLS from domain satisfaction while constraining all coefficients to equality), 48.41% of variance could be explained. Allowing the coefficients of the domain satisfaction ratings to vary (Model 1) again resulted in an improved prediction of the SWLS (*R*^2^ = 51.71%, *R*^2^*_cross-validated_* = 51.54%). Incorporating the main effects of the domain importance ratings (Model 2) again lead to a statistically significant (*p* < .001) change in *R*^2^, with a small gain of 0.23% (*R*^2^ = 52.05%, *R*^2^*_cross-validated_* = 51.77%). Including the importance * satisfaction interactions in the last step (Model 3) again lead to a statistically significant increase in *R*^2^ (*p* < .001), but once again cross-validation led to the conclusion that the difference was completely negligible (*R*^2^ = 52.28%, *R*^2^*_cross-validated_* = 51.85%).

#### Modeling global life satisfaction as a latent factor

Given that global life satisfaction was assessed with five items, it is also possible to model this construct as a latent factor and assess importance weighting in an SEM context. We thus specified a model in which we predicted global life satisfaction (latent factor loading on the five items of the SWLS) from the ten domain satisfaction ratings, the ten domain importance ratings, and the ten satisfaction * importance interactions. This approach also allows us to include respondents with missing values on any of the included items using the full-information maximum likelihood (FIML) estimator, resulting in a sample size of *N* = 5,518.^5^ In Model 0, we restricted the coefficients of all domain satisfaction ratings to equality and set the coefficients of the importance ratings and the satisfaction * importance interactions to 0. In Model 1, we allowed the coefficients of the domain satisfaction ratings to vary. In Model 2, we additionally freed the loadings of the importance ratings, and in Model 3, we finally freed the loadings of the interaction terms. Table 6 summarizes the results of these analyses.

The largest increase in model fit as well as explanation of variance can once again be seen moving from Model 0 to Model 1, when allowing the coefficients of the satisfaction ratings to vary by domain, Δ*R*^2^ = 2.98%, ΔAIC = −275, ΔBIC = −216. When additionally estimating the coefficients of the importance ratings, Model 2, the conventional fit measures CFI, RMSEA and SRMR remain virtually unchanged, and an additional 0.34% or variance in global life satisfaction can be explained. According to the AIC, this model is to be preferred over Model 1, ΔAIC = −14. However, at this point, the BIC already indicates that the more parsimonious Model 1 should be preferred, ΔBIC = 52, as it puts a heftier penalty on the increased complexity given the large sample size.

Likewise, when additionally estimating the coefficients of the interaction terms, fit measures remain virtually unchanged, somewhat more variance in global life satisfaction can be explained (Δ*R*^2^ = 0.27%), which parallels the observation that including the interaction terms in the moderated regression increased the (unadjusted) *R*^2^. Again, according to the AIC, this more complex model is somewhat preferable (ΔAIC = −8), but the BIC again imposes a larger penalty on the added complexity, resulting in a preference for the more parsimonious previous model (ΔBIC = 58). The disagreement between the two information criteria can be explained by the fact that they are trying to answer different questions (as succinctly summarized in Aho, Derryberry, & Peterson, 2014): Whereas BIC seeks to figure out which model is correct in the sense that it might have generated the data (thus performing well in simulations in which data are generated under a relatively simple process), AIC focuses on the best prediction in the context of incompletely specified or infinite parameter models (“All models are wrong, but some are more useful”). Hence, whether or not one interprets these analyses as in favor of importance weighting or not depends on whether one is interested in maximizing predictive performance or finding the true model (assuming that it is finite). In any case, it should be clear that the amount of variance that could be explained by the interaction terms was very small, less than 0.3% *within* the sample.

### Investigating Importance Weighting in Single Domains

To further explore why the importance weighting procedure was not able to account for additional variance in global life satisfaction, we took a close look at the single domains: At least one previous study claimed that importance weighting varies between domains (Tiefenbach & Kohlbacher, 2015).

Inspection of the model coefficients of the moderated regression model including all domains (Table 5, column Model 3) revealed some “counterintuitive” estimates. Two domains indicated statistically significant interactions according to the conventional cut-off of *p* < .05; however, both were negative and thus ran counter to the notion of importance weighting: *b*_housing_interaction_ = −0.04, *p* = .002 and *b*_friend_interaction_ = −0.03, *p* = .002. However, this was not surprising given the data at hand. The satisfaction ratings were correlated across the different domains with values ranging from *r* = .25 (area satisfaction with satisfaction with faith) to .57 (housing satisfaction with area satisfaction; family satisfaction with satisfaction with friends) as can be seen in Figure 1; similarly, the domain importance ratings were intercorrelated (ranging from .07 to .65). Consequently, for example, it was possible to explain about 47% of the variance in satisfaction with friends from the other domain satisfaction ratings. If all domain satisfaction ratings were simultaneously included in the model, and the effect of satisfaction with friends were examined, this would be akin to holding satisfaction in the nine other life domains constant and looking only at the associations between the remaining 53% of the variance and the outcome variable. In other words, the effect of satisfaction with friends reflects *the effect of being more or less satisfied with one's friends than would be predicted on the basis of both satisfaction with and the importance of the other life domains*. When holding everything else constant, the interpretation of the interaction term becomes even more complex.

This reflects the so-called “perils of partialling” (Lynam, Hoyle, & Newman, 2006): The effect of a single predictor variable in a regression model when all other predictor variables are held constant might no longer reflect the actual effect of interest. Thus, the regression model including all domains is not suitable for determining which domains importance weighting does or does not occur in. To further explore differences between the domains, a different analytic procedure is necessary. Thus, we additionally ran moderated regressions in which we predicted the SWLS score from importance, satisfaction, and their interaction, but this time, we investigated only *one domain at a time*. The results from these analyses can be found in Table 7.

Some domains clearly showed the expected interaction pattern. For example, at the average level of job importance, a 1-point increase in job satisfaction predicted a 0.37 point increase in global life satisfaction, *p* < .001. In addition, at the average level of job satisfaction, a 1-point increase in job importance also predicted a (comparably small) 0.04 point increase in global life satisfaction, *p* = .001. A significant positive interaction indicated that for jobs that were rated as more important, the effect of job satisfaction on life satisfaction was more pronounced, *p* < .001. For example, for a person who rated his or her job as 1 point more important than the average, a 1 point increase in job satisfaction was associated with a 0.37 + 0.05 = 0.42 increase in global life satisfaction. The same pattern emerged for satisfaction with marriage (*p*_interaction_ < .001) and satisfaction with faith (*p*_interaction_ < .001). The domains finances and friends also showed this pattern in weaker form and met the conventional threshold for significance, whereas the domains hobbies (*p*_interaction_ = .074) and family (*p*_interaction_ = .091) displayed trends in the same direction that could be labeled “marginally significant” at best. It is noticeable that only one domain—housing— showed a negative interaction (*b* = −0.01, *p* = .529), but it was still very close to zero. Overall, the pattern of results thus suggests that there is some evidence for the expected moderating effect of importance ratings although the strength of this evidence varies across domains. However, as we will show below, it is questionable whether these coefficients should be interpreted as support for a bottom-up weighted model of life satisfaction.

### Is Importance Weighting Domain-Specific?

The high intercorrelation within the importance ratings and within the satisfaction ratings raised the question of whether these interactions are sufficiently specific. For example, it is possible that there is a general satisfaction factor (being satisfied with life domains in general), and a general importance factor (viewing life domains as important in general). Both might reflect either substantial factors, such as personality predispositions, or, in a more mundane interpretation, response biases. The two factors might interact such that individuals who think that life domains per se are important tend to be more strongly affected by their domain satisfaction when judging their overall life satisfaction. Such a general explanation would not go well with a bottom-up weighting approach that implies that the importance that a person ascribes to family (and not, e.g., the importance the person ascribes to health) moderates the effect of family satisfaction on general life satisfaction.

As a first (crude) test of such an alternative explanation, we ran a moderated regression model in which we predicted global life satisfaction from the average satisfaction rating across all domains, the average importance rating across all domains, and the interaction of these two factors. Indeed, in this model, we found a statistically significant (*p* = .005) interaction between the average satisfaction and average importance, albeit with a negative coefficient, which does not support this alternative account. Furthermore, model performance (*R*^2^ = 48.55%) was worse than in the domain-specific moderated regression analyses reported above.

**Figure 2:**
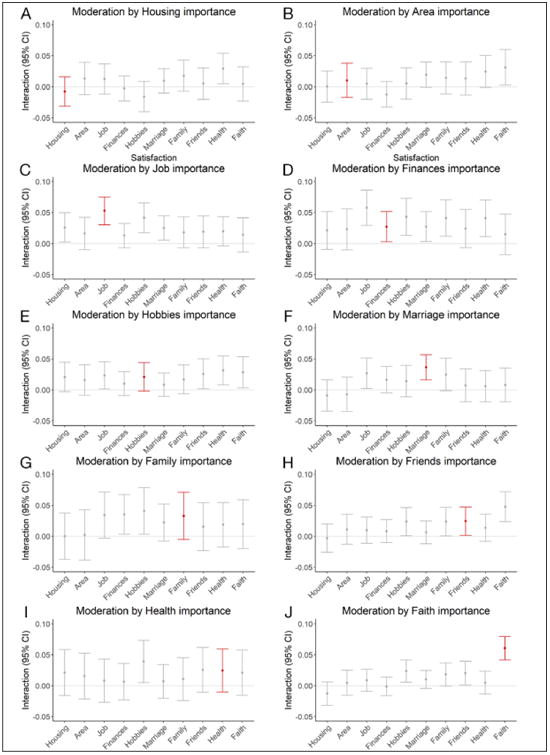
Interaction coefficients from the regression analyses in which each of the 10 importance ratings were combined with each of the 10 satisfaction ratings to predict the SWLS score. Analyses with concordant domains (i.e., combinations for which one would expect a moderating effect of importance ratings) are highlighted in red; *N* = 5,049.

Subsequently, we took a more detailed look at potentially unspecific moderating effects of importance ratings by combining all 10 domain importance ratings with all 10 domain satisfaction ratings and testing their interaction. For example, we ran one regression model in which we predicted the SWLS score from job satisfaction, importance of faith, and their interaction. To support the bottom-up importance-weighted life satisfaction model, the resulting interaction would ideally be smaller than the interaction between job satisfaction and job importance and smaller than the interaction between satisfaction with faith and importance of faith. Figure 2 visually represents the resulting interaction coefficients from the 10 * 10 = 100 separate regression models.

In looking at the overall picture, there was no clear pattern supporting the hypothesis that the importance of specific life domains *distinctly* moderated the association between satisfaction with the respective domain and the SWLS score. Only three domains at least *tended* toward the pattern that could be expected.

First, the coefficient for the interaction between job importance and job satisfaction was the largest of the job importance interactions (Panel C, highlighted). However, the interaction between job importance and hobby satisfaction had a similar magnitude (also Panel C). Furthermore, the effect of job satisfaction was affected by the importance of finances to a similar degree (Panel D).

Second, the coefficient for the interaction between the importance of marriage and marriage satisfaction was the largest of the marriage importance interactions (Panel F, highlighted). However, to a lesser degree, the importance of marriage also potentially moderated the effects of satisfaction in a couple of other life domains (also Panel F). Furthermore, the interaction between marriage satisfaction and importance of finances was comparable in magnitude (Panel D).

Third, the coefficient for the interaction between the importance of faith and satisfaction with faith was the largest of the faith importance interactions (Panel J, highlighted). Importance of friends also moderated the association between the SWLS score and satisfaction with faith (Panel H) but to a lesser extent.

Taken together, only one out of 10 life domains yielded clear support for domain-specific, distinct importance weighting. Note that the results remained almost unchanged when all involved variables were standardized prior to analysis (i.e., when the coefficients reflected β-weights), we provide the corresponding figure on the OSF (https://osf.io/m3ezs/).

## General Discussion

All things considered, our results suggest that importance weighting of domain satisfaction ratings does not improve the prediction of global life satisfaction. Moderated regression analyses including all ten life domains simultaneously indicated that the inclusion of the interactions between importance and satisfaction did not increase the amount of variance explained when taking into account the increased model complexity. Investigating only one domain at a time revealed somewhat more promising results with significant interactions for multiple domains. However, cross-domain analyses suggested that most importance-satisfaction interactions were not domain specific, which is why these significant interactions can hardly be interpreted as evidence for a bottom-up model in which the effect of satisfaction in a specific life domain is modulated by the importance of *that* life domain (and not the importance of a different life domain).

It is worth noting that this lack of support for importance weighting on the individual level aligns with similar findings from another substantive field, the research of self-esteem. In self-esteem research, the idea that one's overall self-evaluation is the importance-weighted average of self-evaluations in specific domains has been popular for more than 100 years (Marsh, 2008), but empirical support has been lacking and methodologically deficient.

Why does importance weighting, despite its intuitive appeal, fail to find support in empirical data? In the following, we will outline possible explanations, which are not mutually exclusive, and their implications for further research.

### Lack of Variability in Importance Ratings

The chance of detecting individual importance weightings is zero if all respondents consider a certain domain to be equally important (see also Schimmack, Diener and Oishi, 2002). For example, in the present study, respondents almost unanimously agreed that a satisfying family life is important, decreasing the chances of finding importance weighting in that domain—if it is important for everyone, the same coefficient should hold for everyone, even if the weighted bottom-up model holds true. In addition, any variation in the importance ratings for domains that are considered unanimously important might not necessarily reflect actual differences in the importance of the respective domains but rather interindividual differences in the usage of the response scale, making it even less likely to detect the desired specific moderating effect of importance ratings.

This explanation is somewhat supported by our findings regarding the domain of faith. For this domain, importance ratings showed the highest variability, and they were also only weakly correlated with importance of other life domains. In line with a weighted bottom-up model, the importance of faith moderated the association between satisfaction with faith and global life satisfaction but not the association between satisfaction with *any other* life domain and global life satisfaction.

Researchers could try to purposefully include life domains that are likely rather unimportant to part of the respondents. For example, one could draw inspiration from the study by Schimmack, Diener and Oishi (2002), which included the performance of the Illini men's basketball team as one “domain of life” with which respondents can be more or less satisfied. Of course, inclusion of such domains is somewhat opposed to the aim of a comprehensive assessment of well-being with as few items as possible, which is often the case in large scale survey studies. As Trauer and Mackinnon (2001) correctly pointed out, well-being measures that include multiple domains are intentionally constructed in a way so that the included domains are relevant for all respondents, making them particularly unsuited to detect the effects of importance weighting.

### Lack of Reliability and/or Validity of Importance Ratings

Campbell et al. (1976, pp. 87–88) already noted that the stability of their importance ratings was lower than those of other measures, raising questions about the reliability of single-item importance measures. Of course, a lack of reliable variance in single item importance measures would also limit their validity and hence undermine chances to detect the effects of importance weighting. This could be solved by the inclusion of more reliable multi-item measures. For example, the SEM approach suggested by Marsh and Scalas (2017) explicitly makes use of multiple importance indicators per domain to estimate a latent importance factor. However, it should also be noted that even using multi-item measures, Marsh and Scalas failed to find evidence for individual importance-weighting for global self-esteem. So at least in that related research domain, a lack of reliability does not seem to be the explanation for the failings of individual importance weighting.

Apart for low reliability, there are other potential reasons for a lack of validity of domain importance ratings. Russel and Hubley (2005) provided a comprehensive list of arguments that have been raised against the validity of importance ratings, including various response biases and simple lack of insight.

### Alternative Moderators of the Association Between Domain and Global Satisfaction

In addition, one could argue that the weights assigned to various life domains when forming a global life satisfaction judgment are not weighted according to importance, but rather according to some other variable. For example, values have been suggested as one moderator (Oishi, Diener, Suh, & Lucas, 1999). Schimmack, Diener and Oishi (2002) stressed the role of accessibility: Only information about life domains that is accessible when the global evaluation is formed can affect it. And on a different level of abstraction, developmental stage might moderate the association, as there is, for example, robust evidence that the effect of income on life satisfaction changes across the life course (Cheung & Lucas, 2015).

### Invalidity of the Individually Weighted Bottom-Up Model

One might also doubt whether individuals do in fact assign varying weights to different life domains when forming their global satisfaction judgment. As Marsh and Scalas (2017) point out, individually weighted models are intuitively very compelling. However, that does of course not imply that they are actually true. Instead, it could be possible that the weights assigned to satisfaction in different life domains are actually homogeneous across respondents, in line with normative weighting. This could potentially be tested without further consideration of the points mentioned above (psychometric features of importance ratings, suitability of importance as moderator) by taking a repeated-measures approach and assessing relationships between domain satisfaction and global satisfaction judgments *within* subjects. In such a study, it should be possible to detect interindividual differences in domain weighting, assuming that such differences are somewhat stable (see e.g. Schimmack & Oishi, 2005, for evidence that chronically accessible information seems more important than temporarily accessible information).

### Using the Wrong Criterion

So far, we have assumed that it is sensible to evaluate the validity of individual importance weighting by using a global life satisfaction measure as criterion. The validity of global life satisfaction measures has been extensively illustrated by countless plausible associations with, for example, non-self-report measures and life circumstances (Diener, Inglehart, & Tay, 2013). However, that does not imply that global life satisfaction judgments necessarily reflect an *optimal* (or optimally weighted) assessment— people might assign exaggerated weights to certain life domains (e.g., income; Kahneman, Krueger, Schkade, Schwarz, & Stone, 2006) and underestimate the weight of others. Thus, it could be promising to evaluate importance weighting against alternative criterion variables (as in e.g., Marsh & Scalas, 2017; see Russell & Hubley, 2005, for a similar argument) in the same way in which global life satisfaction judgments have been evaluated, and even to compare the performance of weighted composite scores against those of global judgments. In fact, there is some empirical evidence that domain satisfaction judgments (without individual weighting) are approximately as valid as global life-satisfaction judgments (Zou, Schimmack, & Gere, 2013). Hence, it might be misguided to rely on global measures to assess the validity of importance weighting.

## Figures and Tables

**Table 1:** Exact Wording of the Domain Importance and the Domain Satisfaction Items in the Panel Study of Income Dynamics Well-Being Supplement.

Domain abbreviation	Domain importance item	Domain satisfaction item
	Below is a list of things that may or may not be important to you. How important are each of the following to you?	How satisfied are you with each of the following?
Housing	Living in a house or apartment that I like	My house or apartment
Area	Living in a city or place that I like	The city or place that I live in
Job	Having an interesting job	My job
Finances	Being financially secure or not having to worry about money	My financial situation
Hobbies	Having hobbies or things that I like to do outside of work	My hobbies
Marriage	Having a happy marriage or romantic relationship	My marriage or romantic relationship
Family	Having a good family life	My family life
Friends	Having good friends	My friendships
Health	Being in good health	My health
Faith	Having a strong religious faith	My faith

*Note*. Domain abbreviations are used throughout the manuscript to refer to the respective life domains.

**Table 2:** Descriptive Statistics and Correlations with the Satisfaction With Life Scale of the Domain Importance Ratings in the PSID (*N* = 5,049).

Domain	Importance rating (% of sample)					*M*	*SD*	Correlation with SWLS

	0	1	2	3	4			
Housing	0.50	1.72	13.90	44.94	38.94	3.20	0.78	.163
Area	0.48	1.51	12.10	46.92	39.00	3.22	0.75	.178
Job	0.79	1.78	16.30	45.99	35.14	3.13	0.80	.134
Finances	0.14	0.53	8.16	38.60	52.56	3.43	0.68	.004
Hobbies	0.46	2.81	20.20	44.37	32.16	3.05	0.82	.139
Marriage	0.69	1.15	7.03	28.38	62.75	3.51	0.74	.203
Family	0.04	0.38	2.22	25.85	71.52	3.68	0.54	.184
Friends	0.53	2.69	17.57	38.66	40.54	3.16	0.84	.166
Health	0.12	0.22	3.19	30.94	65.54	3.62	0.57	.113
Faith	3.21	6.67	14.87	25.27	49.97	3.12	1.09	.100

Response scale: 0 = not at all important, 1 = a little important, 2 = somewhat important, 3 = very important, 4 = extremely important.

**Table 3:** Descriptive Statistics and Correlations with the Satisfaction With Life Scale of the Domain Satisfaction Ratings in the PSID (*N* = 5,049).

Domain	Satisfaction rating (% of sample)					*M*	*SD*	Correlation with SWLS

	0	1	2	3	4			
Housing	3.60	5.76	27.09	42.92	20.62	2.71	0.97	.497
Area	2.67	5.88	24.90	43.91	22.64	2.78	0.95	.422
Job	5.37	8.46	31.17	37.41	17.59	2.53	1.04	.473
Finances	12.66	11.51	37.04	28.66	10.14	2.12	1.14	.589
Hobbies	4.54	10.93	32.62	36.98	14.93	2.47	1.02	.450
Marriage	7.63	6.85	17.90	34.09	33.53	2.79	1.20	.522
Family	1.74	3.43	17.49	41.59	35.75	3.06	0.91	.555
Friends	2.16	6.75	25.65	41.67	23.77	2.78	0.95	.454
Health	4.38	6.85	29.83	39.37	19.57	2.63	1.01	.457
Faith	1.49	6.67	22.24	36.09	33.51	2.93	0.98	.318

Response scale: 0 = not at all satisfied, 1 = a little satisfied, 2 = somewhat satisfied, 3 = very satisfied, 4 = completely satisfied.

**Table 4:** Intercorrelations between the different composite scores of domain satisfaction items and the Satisfaction With Life Scale (SWLS) in the PSID (*N* = 5,049).

	SWLS	Individually w. composite	Normatively w. composite
Simple sum score	.695	>.999	.993
Normatively w. composite	.698	.993	
Individually w. composite	.699		

**Table 5:** Results of Multiple Regression Analyses Predicting the Satisfaction With Life Scale (SWLS) score from Satisfaction and Importance across All Domains.

Predictor		Model 1		Model 2		Model 3	

		*b*	*p*	*b*	*p*	*b*	*p*
Housing	Satisfaction	0.09	<.001	0.09	<.001	0.09	<.001
	Importance			0.01	.396	0.01	.725
	Interaction					−0.04	.002
Area	Satisfaction	0.03	.013	0.02	.056	0.02	.041
	Importance			0.04	.016	0.03	.027
	Interaction					−0.03	.805
Job	Satisfaction	0.08	<.001	0.08	<.001	0.08	<.001
	Importance			0.01	.517	0.01	.325
	Interaction					0.02	.055
Finances	Satisfaction	0.18	<.001	0.18	<.001	0.18	<.001
	Importance			− 0.06	<.001	−0.05	<.001
	Interaction					0.02	.124
Hobbies	Satisfaction	0.02	.040	0.02	.079	0.02	.062
	Importance			0.00	.878	0.00	.902
	Interaction					0.00	.972
Marriage	Satisfaction	0.11	<.001	0.10	<.001	0.10	<.001
	Importance			0.03	.076	0.03	.071
	Interaction					0.01	.303
Family	Satisfaction	0.17	<.001	0.17	<.001	0.17	<.001
	Importance			0.01	.579	0.02	.430
	Interaction					0.01	.444
Friends	Satisfaction	0.03	.020	0.04	.003	0.03	.012
	Importance			−0.02	.098	−0.03	.016
	Interaction					−0.03	.002
Health	Satisfaction	0.08	<.001	0.09	<.001	0.09	<.001
	Importance			−0.04	.048	−0.03	.087
	Interaction					0.01	.666
Faith	Satisfaction	0.00	.816	0.00	.776	0.00	.714
	Importance			0.00	.924	0.00	.942
	Interaction					0.00	.793

*R* ^2^*_in sample_*(and Δ to previous model)		51.63%		51.94% (+0.31%)		52.20% (+0.26%)	
*R^2^_cross-validated_* (Δ)		51.15%		51.22% (+0.07%)		51.23% (+0.01%)	

*Note*. *N* = 5,049. Predictors were centered before the interaction terms were calculated (Interaction = Satisfaction * Importance).

**Table 6:** Results of SEM Analyses Predicting Global Life Satisfaction from Domain Satisfaction, Domain Importance, and their Interaction (*N* = 5,518).

	Model 0	Model 1	Model 2	Model 3
*Fit measures*				

CFI	.963	.977	.978	.979
RMSEA	.030	.024	.025	.025
SRMR	.017	.009	.009	.009
*Information criteria*				

AIC	428769	428494	428480	428472
BIC	428875	428659	428711	428769
*Variance of global life satisfaction explained*				

*R*^2^	53.08%	56.06%	56.40%	56.67%

**Table 7:** Results of Independent Multiple Regression Analyses Predicting the Satisfaction With Life Scale (SWLS) from Satisfaction and Importance Separately for Each Domain.

Domain	Domain importance		Domain satisfaction		Importance *satisfaction	

	*b*	*p*	*b*	*P*	*B*	*p*
Housing	0.06	<.001	0.42	<.001	−0.01	.529
Area	0.09	<.001	0.35	<.001	0.01	.464
Job	0.04	.001	0.37	<.001	0.05	<.001
Finances	0.00	.966	0.43	<.001	0.03	.030
Hobbies	−0.03	.023	0.38	<.001	0.02	.074
Marriage	0.02	.171	0.37	<.001	0.04	<.001
Family	0.00	.963	0.51	<.001	0.03	.091
Friends	−0.04	.012	0.41	<.001	0.02	.037
Health	0.02	.290	0.37	<.001	0.02	.166
Faith	−0.04	.001	0.31	<.001	0.06	<.001

*Note*. *N* = 5,049. Each line in the table represent an independent regression model. We centered the predictors before we calculated the interaction terms (Interaction = Satisfaction * Importance).

## Footnotes

1 Note that the authors claimed that the results they obtained were not invariant under a linear transformation of “one or both elements of the composite” (Trauer & Mackinnon, 2001, p. 581). However, this claim is not correct. If the satisfaction ratings are linearly transformed before the weighted composite score is calculated, the resulting score is a linear transformation of the weighted composite score of the untransformed satisfaction ratings. Thus, a linear transformation of the satisfaction ratings will not affect, for example, correlations between the composite score and other variables. But this does not hold for a linear transformation of the importance ratings because they are located in the denominator of the formula of the composite score.

2 Note that, as mentioned earlier, the weighted composite scores are not invariant under linear transformations of the data. Setting *not at all* to zero captures the idea that if a respondent says a domain does not matter at all, it is not incorporated into the weighted composite. However, we additionally ran all analyses with a different coding scheme (1 = *not at all important*, 2 = *a little important*, 3 = *somewhat important*, 4 = *very important*, 5 = *extremely important*) and arrived at similar results.

3 Note that the degrees of freedom differed depending on whether the scores were standardized or not. Without standardization, it was not possible to calculate profile correlations for all respondents because some respondents gave the same reply to all importance items/all satisfaction items, and this made it impossible to calculate correlations. In addition, the profile correlation was 1 for some respondents, and these correlations resulted in infinite Fisher *z*-scores that had to be excluded before averaging. These problems no longer occurred after the variables were standardized.

4 Furthermore, when comparing to the model *only* including domain satisfaction, it becomes clear that even in combination, the importance ratings and their interactions with satisfaction only contribute a completely negligible increase in *R*^2^ of less than 0.1%.

5 Notice that we excluded respondents who reported that the domains partner, job, or faith did not apply, because these missings are conceptually different from missing due to nonresponse.

## References

[R1] Aho K, Derryberry D, Peterson T (2014). Model selection for ecologists: the worldviews of AIC and BIC. Ecology.

[R2] Allik J, Borkenau P, Hřebíčková M, Kuppens P, Realo A (2015). How are personality trait and profile agreement related?. Frontiers in Psychology.

[R3] Bischl B, Lang M, Kotthoff L, Schiffner J, Richter J, Studerus E, Casalicchio G, Jones Z (2016). mlr: Machine Learning in R. Journal of Machine Learning Research.

[R4] Campbell A, Converse PE, Rodgers WL (1976). The quality of American life: Perceptions, evaluations, and satisfactions.

[R5] Cheung F, Lucas RE (2015). When does money matter most? Examining the association between income and life satisfaction over the life course. Psychology and Aging.

[R6] Diener Ed, Emmons RA, Larsen RJ, Griffin S (1985). The satisfaction with life scale. Journal of Personality Assessment.

[R7] Diener Ed, Inglehart R, Tay L (2013). Theory and validity of life satisfaction scales. Social Indicators Research.

[R8] Freedman VA (2017). The Panel Study of Income Dynamics' Well-being and Daily LifeSupplement (PSID-WB) User Guide: Final Release 1.

[R9] George LK, Okun MA, Landerman R (1985). Age as a moderator of the determinants of life satisfaction. Research on Aging.

[R10] Hsieh CM (2003). Counting importance: The case of life satisfaction and relative domain importance. Social Indicators Research.

[R11] Hsieh CM (2012). Should we give up domain importance weighting in QoL measures?. Social Indicators Research.

[R12] Hsieh CM (2016). Domain importance in subjective well-being measures. Social Indicators Research.

[R13] Kahneman D, Krueger AB, Schkade D, Schwarz N, Stone AA (2006). Would you be happier if you were richer? A focusing illusion. Science.

[R14] Lucas RE (2004). Top-down and bottom-up models of life satisfaction judgments. Paper presented at the 6th International German Socio-Economic Panel Study User Conference.

[R15] Lynam DR, Hoyle RH, Newman JP (2006). The perils of partialling: Cautionary tales from aggression and psychopathy. Assessment.

[R16] Marsh HW (2008). The Elusive Importance Effect: More Failure for the Jamesian Perspective on the Importance of Importance in Shaping Self-Esteem. Journal of Personality.

[R17] Marsh HW, Scalas LF (2017). Individually Weighted-Average Models: Testing a Taxonomic SEM Approach Across Different Multidimensional/Global Constructs Because the Weights “Don't Make No Nevermind”. Structural Equation Modeling: A Multidisciplinary Journal.

[R18] Oishi S, Diener EF, Lucas RE, Suh EM (1999). Cross-cultural variations in predictors of life satisfaction: Perspectives from needs and values. Personality and Social Psychology Bulletin.

[R19] Oishi S, Diener E, Suh E, Lucas RE (1999). Value as a moderator in subjective well-being. Journal of Personality.

[R20] Philip EJ, Merluzzi TV, Peterman A, Cronk LB (2009). Measurement accuracy in assessing patient's quality of life: to weight or not to weight domains of quality of life. Quality of Life Research.

[R21] R Core Team (2017). R: A language and environment for statistical computing.

[R22] Revelle W (2016). psych: Procedures for Personality and Psychological Research.

[R23] Rosseel Y (2012). lavaan: An R Package for Structural Equation Modeling. Journal of Statistical Software.

[R24] RStudio Team (2016). RStudio: Integrated Development for R.

[R25] Russell LB, Hubley AM (2005). Importance ratings and weighting: Old concerns and new perspectives. International Journal of Testing.

[R26] Russell LB, Hubley AM, Palepu A, Zumbo BD (2006). Does weighting capture what's important? Revisiting subjective importance weighting with a quality of life measure. Social Indicators Research.

[R27] Schimmack U, Diener E, Oishi S (2002). Life-satisfaction is a momentary judgment and a stable personality characteristic: The use of chronically accessible and stable sources. Journal of Personality.

[R28] Schimmack U, Oishi S (2005). The influence of chronically and temporarily accessible information on life satisfaction judgments. Journal of Personality and Social Psychology.

[R29] Steiger JH (1980). Tests for comparing elements of a correlation matrix. Psychological Bulletin.

[R30] Survey Research Center, Institute for Social Research, University of Michigan, Ann Arbor (2017). Panel Study of Income Dynamics, public use dataset.

[R31] Tiefenbach T, Kohlbacher F (2015). Individual differences in the relationship between domain satisfaction and happiness: The moderating role of domain importance. Personality and Individual Differences.

[R32] Trauer T, Mackinnon A (2001). Why are we weighting? The role of importance ratings in quality of life measurement. Quality of Life Research.

[R33] Wickham H (2007). Reshaping Data with the reshape Package. Journal of Statistical Software.

[R34] Wickham H (2009). ggplot2: Elegant Graphics for Data Analysis.

[R35] Wickham H (2016). tidyr: Easily Tidy Data with ‘spread()’ and ‘gather()’ Functions R package version 0.5.0.

[R36] Wickham H, Francois R (2016). dplyr: A Grammar of Data Manipulation R package version 0.5.0.

[R37] Wu CH (2008). Can we weight satisfaction score with importance ranks across life domains?. Social Indicators Research.

[R38] Wu CH, Yao G (2006). Do we need to weight satisfaction scores with importance ratings in measuring quality of life?. Social Indicators Research.

[R39] Yarkoni T, Westfall J (2017). Choosing prediction over explanation in psychology: Lessons from machine learning. Perspectives on Psychological Science.

[R40] Zou C, Schimmack U, Gere J (2013). The validity of well-being measures: A multiple-indicator–multiple-rater model. Psychological Assessment.

